# Zanubrutinib: past, present, and future

**DOI:** 10.1038/s41408-023-00902-x

**Published:** 2023-09-11

**Authors:** Constantine S. Tam, Javier L. Muñoz, John F. Seymour, Stephen Opat

**Affiliations:** 1 Alfred Hospital and Monash University, Melbourne, VIC Australia; 2 Mayo Clinic, Phoenix, AZ USA; 3 Peter MacCallum Cancer Centre, Royal Melbourne Hospital & University of Melbourne, Melbourne, VIC Australia; 4 Monash Health and Monash University, Clayton, VIC Australia

**Keywords:** Drug development, Drug development

## Abstract

In recent years, Bruton tyrosine kinase (BTK) inhibitors have provided significant advances in the treatment of patients with B-cell malignancies. Ibrutinib was the first BTK inhibitor to be approved, and it changed the standard-of-care treatment for diseases such as chronic lymphocytic leukemia, mantle cell lymphoma, marginal zone lymphoma, and Waldenström macroglobulinemia, improving efficacy outcomes and safety compared to chemotherapy. In this article, we review the development of zanubrutinib, a next-generation BTK inhibitor, from molecular design to patient-related outcomes. We start this journey by providing insights into the discovery of BTK and the physiologic, genetic, and molecular characterization of patients lacking this kinase, together with the brief treatment landscape in the era of chemo-immunotherapies. Zanubrutinib was originally developed by applying a structure-activity strategy to enhance the specificity as well as enzymatic and pharmacokinetic properties. Preclinical studies confirmed greater specificity and better bioavailability of zanubrutinib compared with that of ibrutinib, which supported the initiation of clinical trials in humans. Preliminary clinical results indicated activity in B-cell malignancies together with an improved safety profile, in line with less off-target effects described in the preclinical studies. The clinical program of zanubrutinib has since expanded significantly, with ongoing studies in a wide range of hemato-oncological diseases and in combination with many other therapies. Zanubrutinib currently is approved for various B-cell malignancies in multiple countries. This story highlights the importance of multidisciplinary collaborative research, from bench to bedside, and provides an example of how the commitment to finding improved treatment options should always run parallel to patient care.

## Introduction

B-cell malignancies are the most frequent hematologic cancers and include a heterogeneous group of more than 40 malignancies caused by the uncontrolled proliferation of B-cells [1]. Not only are they the hematologic cancer most frequently diagnosed globally, with 544,000 cases of non-Hodgkin lymphoma in 2020, but they are also associated with considerable morbidity, with 260,000 deaths reported worldwide in 2020 [2].

**Fig. 1:**
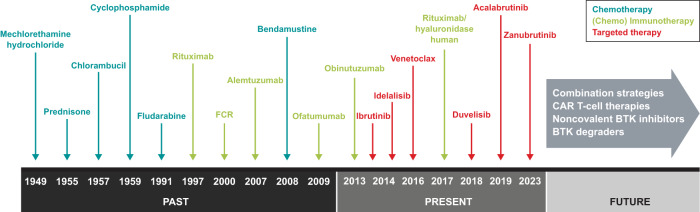
Treatment evolution in chronic lymphocytic leukemia [30, 95, 96]. CAR chimeric antigen receptor, BTK Bruton tyrosine kinase, FCR fludarabine, cyclophosphamide, and rituximab.

Diagnostic tools to identify and classify B-cell malignancies have improved the cytologic, molecular, and genetic understanding of each specific disease, thereby also permitting the development of improved therapies for each individual malignancy. In the past few decades, therapies for B-cell malignancies have evolved considerably. A brief overview of chronic lymphocytic leukemia (CLL) is illustrative. Until the 1980s, cytotoxic agents, including chlorambucil and cyclophosphamide, were the only available therapeutic options. The development of the purine nucleoside analogue fludarabine in the 1990s and its use in various combinations helped enhance treatment outcomes (Fig. 1). Despite improvements in response, duration of remission, and progression-free survival (PFS), increases in overall survival (OS) were limited [3]. Furthermore, chemotherapy was associated with hematologic toxicity, secondary cancers such as myelodysplastic syndromes and acute myeloid leukemia, and other adverse effects [4, 5].

Better understanding of the cellular receptor pathways involved in malignant B-cells led to development of monoclonal antibodies targeting key surface antigens and receptors involved in the survival and proliferation of malignant cells, such as the anti-cluster of differentiation 20 (CD20) antibody rituximab [6]. Rituximab combined with fludarabine and cyclophosphamide (FCR) notably improved survival [7, 8], but this combination primarily was used in fit patients because it was too toxic (i.e., hematologic toxicity, risk of infections) for frail and/or older patients [9]. Rituximab in combination with bendamustine (BR), although not as effective as FCR in younger patients [10], is associated with fewer and less severe toxic effects and, thus, became the preferred regimen in frailer patients [9]. Improvements in allogeneic stem cell transplantation offered a potentially curative option, but only for young patients [5, 11].

**Fig. 2:**
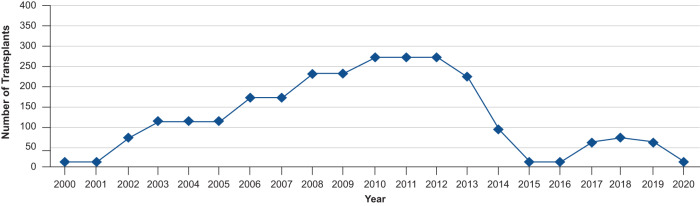
Number of allogeneic transplantations performed in adult patients with CLL in the US during 2000 to 2020. Reductions in the number of transplantations in patients with CLL can be observed from 2013 [12]. CLL chronic lymphocytic leukemia.

Interestingly, in recent years, the number of allogeneic stem cell transplantations performed in patients with CLL has decreased considerably. The development and the use of targeted therapies, including Bruton tyrosine kinase (BTK) inhibitors, may have contributed to this reduction (Fig. 2) [12].

## The discovery of Bruton tyrosine kinase

The history of BTK started with the first diagnosis and description of a disease presenting with absence of mature B-cells and immunoglobulin G, and characterized by recurrent bacterial infections; this was X-linked agammaglobulinemia (XLA) described by Dr Ogden Bruton and named eponymously after him [13]. Subsequent genetic characterization of XLA revealed that it was caused by the lack of expression of BTK, a tyrosine kinase of the Tec-family, due to mutations in a gene located on the X chromosome [14, 15]. BTK is essential for maturation of pre–B cells and other processes related to B-cell physiology, as shown by characterization of *Btk*-null mice [16] and by the study of more than 800 mutations in the *BTK* gene in patients with XLA [17]. Extensive molecular and cellular analyses have confirmed the critical role of BTK in multiple hematopoietic signals, which go beyond the B-cell antigen receptor pathway, and initial inhibitory agents showed preliminary activity as antileukemic agents, setting up BTK as a potential target in B-cell malignancies [18].

### Rationale for and development of ibrutinib, the first-generation Bruton tyrosine kinase inhibitor

Further understanding of the oncogenic dependencies of B-cell malignancies expanded the potential therapeutic targets. This opened the possibility of obtaining durable disease control with more narrowly targeted therapies, with an improved safety profile, enabling broader application to more patient subgroups [19]. Several newer therapeutic targets—such as CD37, spleen-associated tyrosine kinase (Syk), phosphoinositide 3-kinase (PI3Kδ), CD19, myeloid cell leukemia 1 (MCL1), and B-cell lymphoma 2 (BCL-2) [11], receptor tyrosine kinase-like orphan receptor 1 (ROR1)—have been studied in lymphoid cancers; however, targeting BTK has proven to be one of the most successful strategies for management of B-cell malignancies owing to broad efficacy across a range of diseases, safety, and dosing convenience of oral administration. BTK is an essential component of the B-cell receptor intracellular signaling pathway, mediating B-cell development, proliferation, and survival [20]. Aberrant BTK signaling plays a critical role in the development of various B-cell malignancies including diffuse large B-cell lymphoma [21], CLL [22], mantle cell lymphoma (MCL) [23, 24], Waldenström macroglobulinemia (WM) [25, 26], and marginal zone lymphoma (MZL) [27].

The first-generation BTK inhibitor ibrutinib was initially synthesized in 2007 and described as an irreversible BTK inhibitor with potential therapeutic value in rheumatoid arthritis [28]. Clinical studies in CLL [29–34], MCL [35], MZL [27], and WM [36] subsequently showed benefits in these patients. Approval of ibrutinib [37] by the United States Food and Drug Administration (FDA) in 2013 changed the treatment paradigm of various hematologic malignancies, and ibrutinib rapidly became the standard of care for treating patients with certain subtypes of non-Hodgkin lymphoma and CLL [38, 39]. Not only did treatment standards change with the introduction of ibrutinib but also clinical endpoints needed to be redefined. For example, ibrutinib causes an initial mobilization of CLL cells to the peripheral blood; this paradoxical cellular redistribution was initially mistaken for progressive disease, but the reduction in lymphadenopathy and improvement in cytopenias occurring in parallel suggests that these effects are manifestations of response to the treatment. Considering these unexpected effects of ibrutinib, isolated progressive lymphocytosis would not necessarily be considered a sign of disease progression unless there is other evidence of progressive disease [40].

Despite the considerable improvement in outcomes and quality of life in patients treated with ibrutinib, various adverse events hamper its use (i.e., atrial fibrillation and ventricular dysrhythmias, hypertension, bleeding, rash, and diarrhea). These adverse events lead to treatment discontinuation in up to 23% of patients in clinical studies and up to 49% of patients in community practices [41]. Most of these adverse events are not observed in patients with XLA and congenital deficiency of BTK [13], and thus it was hypothesized that they may be related to off-target activity of the kinase inhibitor. Later studies [42] showed that ibrutinib binding to c-terminal Src kinase may be related to atrial fibrillation [43], inhibition of Tec-family kinases may be related to bleeding events [44], and inhibition of the epidermal growth factor receptor may be related to rash and diarrhea [45]. Moreover, comparison of changes in biomarkers among healthy patients, patients with XLA, and patients with CLL treated with ibrutinib revealed an increase in 6 biomarkers related to atrial fibrillation in a B-cell–independent manner in patients treated with ibrutinib, but not in those with XLA [46]. This evidence suggests that the broad kinome profile and off-target inhibition of ibrutinib may be related to many of these adverse events [42].

After the initial enthusiasm of ibrutinib, additional preclinical studies and long-term clinical results provided evidence for certain aspects that could be improved. Adverse events related to off-target inhibition, primary and secondary resistances, and long-term administration [47] highlighted the need to develop agents that could build upon the successful outcomes of ibrutinib. Development of various second-generation BTK inhibitors (e.g., acalabrutinib and zanubrutinib) was initiated to overcome the limitations of ibrutinib.

## Development of a next-generation Bruton tyrosine kinase, zanubrutinib

**Fig. 3:**
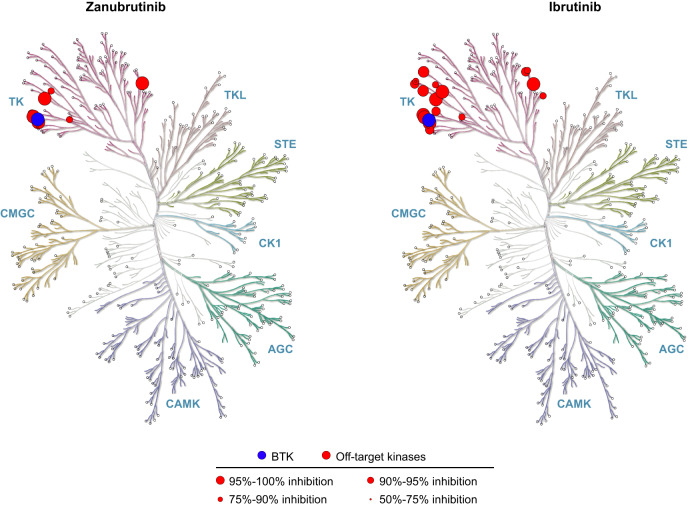
Kinase selectivity of zanubrutinib and ibrutinib [49]. BTK Bruton tyrosine kinase.

The BTK development program at BeiGene (San Mateo, California, USA; and Shanghai, China) began in 2012, with the multidisciplinary collaboration between the medical, biochemistry, discovery biology, and in vivo pharmacology departments at BeiGene in China. This team screened more than 3000 compounds in 2013 to find the molecule with the highest therapeutic potential: BGB-3111 (the 3111th compound screened), later named zanubrutinib [48]. The chemical design of zanubrutinib was guided by a structure-activity strategy to enhance specificity for BTK, minimize off-target binding and associated toxicities, and improve pharmacokinetic properties [48]. Zanubrutinib showed greater selectivity versus other kinases during profile assessment of 370 kinases (Fig. 3) [49], as well as potent inhibitory activity against BTK; zanubrutinib demonstrated more than 50% inhibition in seven kinases, whereas ibrutinib demonstrated more than 50% inhibition in 17 kinases other than BTK (Table 1). Ibrutinib has active metabolites with twofold higher systemic exposure than the parent molecule. Although 1 of these active metabolites (PCI-45227) is 15-fold less potent against BTK compared with the ibrutinib parent molecule, the metabolite still has some activity for kinases other than BTK, which may contribute to off-target toxicities. In contrast, despite zanubrutinib undergoing extensive metabolism (primarily via a cytochrome P450, family 3, subfamily A [CYP3A]-mediated pathway), no active metabolites were detected in the circulation [50]. The most abundant metabolite in the plasma is the inactive mono-hydroxylate of the phenoxy phenyl ring (BGB-7941), which represents less than 10% of the total drug concentration in the circulation and is not considered to contribute significantly to the effects of zanubrutinib [50, 51].

**Table 1:** Kinase profiling at concentrations of 100 × IC_50_ based on BTK IC_50_.

Targets with more than 50% inhibition are highlighted in red (RBC kinase panel, 1 μM ATP).

*ABL2* Abelson tyrosine-protein Kinase, *ARG* ABL-related gene, *ATP* adenosine triphosphate*, AXL* anexelekto*, BLK* B lymphocyte kinase, *BMPR2* bone morphogenetic protein receptor type 2, *BMX* bone marrow X kinase, *BRK* breast tumor kinase, *BTK* Bruton tyrosine kinase, *c-Src* cellular sarcoma virus transforming gene kinase, *CSK* C-terminal Src kinase, *EGFR* epidermal growth factor receptor, *ERBB4* Erb-B2 receptor tyrosine kinase 4, *ERN1/IRE1* endoribonuclease inositol-requiring enzyme 1, *ETK* epithelial and endothelial tyrosine kinase, *FGR* fetal growth restriction, *FLT3* fms-like tyrosine kinase 3, *FRK* Fyn-related kinase, *HCK* hematopoietic cell kinase, *HER2* human epidermal growth factor receptor 2, *HER4* human epidermal growth factor receptor 4, *IC*_*50*_ 50% inhibitory concentration*, ITK* interleukin-2-inducible T-cell kinase*, JAK3* Janus kinase 3, *LBK* lymphocyte-specific protein tyrosine kinase, *LCK* lymphocyte-specific protein tyrosine kinase, *LYN* lck/Yes-related novel protein tyrosine kinase, *MEKK1* mitogen-activated protein kinase 1, *MKNK2* MAPK interacting serine/threonine kinase-2, *MSK2* mitogen- and stress-activated protein kinase-2, *PRKCD* protein kinase C delta, *PTK5* protein tyrosine kinase 5, *RBC* red blood cell, *RPS6KA4* ribosomal protein S6 kinase, *SRMS* Src-related kinase lacking C-terminal regulatory tyrosine and N-terminal myristoylation sites, *STK33* serine/threonine kinase 33, *TEC* thymic epithelial cells*, TXK* TXK tyrosine kinase, *WNK1* with-no-lysine protein kinase, *YES* Y73 and Esh sarcoma kinase.

**Fig. 4:**
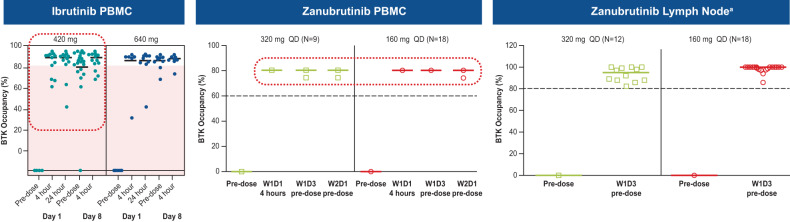
Zanubrutinib BTK occupancy in peripheral blood mononuclear cells and in lymph nodes by dose regimens relative to those in ibrutinib [52, 54, 97]. ^a^The clinical significance of having high BTK occupancy in lymph nodes is unknown. BTK Bruton tyrosine kinase, D day, PBMC peripheral blood mononuclear cell, QD once daily, W week.

**Fig. 5:**
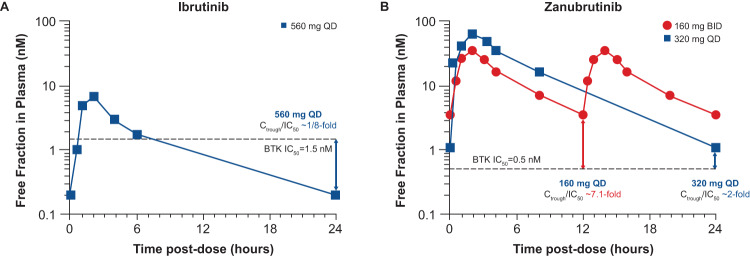
Concentration-time profiles. Values are shown for **A** ibrutinib and **B** zanubrutinib [50, 52, 56]. BID twice daily, BTK Bruton tyrosine kinase, C_trough_ predose trough concentration, IC_50_ half-maximal inhibitory concentration, QD once daily.

Zanubrutinib achieved 100% peripheral blood BTK blockade at a dose of 40 mg daily, and the clinical dose was optimized to achieve 94% and 100% BTK occupancies in lymph nodes, as proven by biopsy results, at the approved doses of 320 mg once daily (QD) or 160 mg twice daily (BID), respectively [52, 53]. In comparison, ibrutinib showed more than 90% blood BTK occupancy at the approved dose of 420 mg QD; however, in some patients BTK occupancy in peripheral blood mononuclear cells fell below 80% (Fig. 4), and systematic evaluation of deep tissue BTK blockade was not performed on the dose-finding studies of ibrutinib [54, 55]. While high levels of peripheral blood BTK occupancy are seen with several agents, zanubrutinib’s high plasma levels may enable penetration into lymph nodes and other niches (i.e., bone marrow) which could account for the improved efficacy of zanubrutinib over ibrutinib in randomized phase 3 studies in CLL and WM. Another key attribute resulting from structural differences between zanubrutinib and ibrutinib is the higher bioavailability of zanubrutinib and its ability to achieve sustained therapeutic exposure, which may directly affect efficacy. At the approved dose, ibrutinib concentration decreases below the half-maximal inhibitory concentration (IC_50_) level at 6 h after dose administration, whereas zanubrutinib concentration remains above the IC_50_ level at all times with both approved doses. In addition, the area-under-the-curve of zanubrutinib is approximately eight times higher than that of ibrutinib at a dose of 560 mg QD (Fig. 5) [50, 52, 56]. The steady-state exposures of zanubrutinib enable deep and durable BTK inhibition in peripheral blood mononuclear cells and lymph nodes, including any newly synthesized BTK molecules [52].

Zanubrutinib has a considerably improved drug-drug interaction profile compared with ibrutinib. Drug-drug interaction studies showed that zanubrutinib, in contrast to ibrutinib, could be administered with CYP3A inhibitors by reducing the dose to 80 mg QD with strong inhibitors, and 80 mg BID with moderate inhibitors; no dose reduction was needed for coadministration of mild CYP3A inhibitors [57, 58]. These improvements in pharmacokinetic properties allowed use in a broader spectrum of patients, and having the option of BID and QD dosing schedules and pills of 80 mg allow for a more convenient administration. Because extrinsic factors do not affect the bioavailability, zanubrutinib can be administered with or without food [57]. The improved drug-drug interaction profile allows zanubrutinib administration concomitantly with proton pump inhibitors, direct oral anticoagulants, warfarin, and other medications relevant for treating B-cell malignancies, if clinically desirable to do so. Patients 65 years and older with CLL frequently are under treatment with anticoagulants, which increases the risk of bleeding. The fact that zanubrutinib can be safely administered concomitantly with anticoagulants without prohibitive risk of bleeding is important for patient management [59]. In addition, the pharmacokinetic profile of zanubrutinib is not directly affected by patient characteristics such as ethnicity [60], or concomitant hepatic or renal impairment. Patients with mild or moderate hepatic impairment do not require dose modifications; in patients with severe hepatic impairment, the dose is reduced to 80 mg BID. For patients with renal impairment, no dose modifications are required [57, 58].

### Clinical development of zanubrutinib

Not long after the preclinical characterization of zanubrutinib, a decision was made for clinical development of the drug in Australia owing to the country’s favorable regulatory environment and rapid clinical research start-up capability. On July 15, 2013, a meeting was held in Melbourne, Australia, with professors Constantine Tam, Andrew Roberts, John Seymour, Andrew Grigg, and Stephen Opat. This Australian meeting brought together researchers with experience in BTK inhibitors and institutions with the capacity to conduct phase 1, first-in-human studies. After review of the preclinical data, a trial design, sketched on a napkin, rapidly evolved into the formal study protocol, and 6 months later, on August 25, 2014, the first patient received a dose of zanubrutinib.

This phase 1 study (NCT02343120) included 17 patients with B-cell malignancies in the dose-escalation part and 94 patients with CLL/small lymphocytic lymphoma (SLL) in the cohort-expansion part. The preliminary results from this study were presented at the 2015 American Society of Hematology Annual Meeting and highlighted the potent efficacy, improved pharmacokinetic properties, and promising tolerability even at higher doses of zanubrutinib [53, 61]. Prompted by these early data, a comprehensive development program for zanubrutinib was organized.

The first clues to zanubrutinib being a potentially superior drug to ibrutinib came from the observation of unexpectedly high very good partial response (VGPR) rates in patients with WM in the phase 1 study. Additionally, patients who had sequential intra-patient escalation of zanubrutinib above the 80 mg daily dose (equivalent to ibrutinib 560 mg) showed progressive improvement in their immunoglobulin M response, which suggested that the level of BTK inhibition could be further optimized in WM. Furthermore, the early investigators found that the rate of atrial fibrillation appeared to be lower than anticipated for the population treated.

## The present: approved indications and current status

**Fig. 6:**
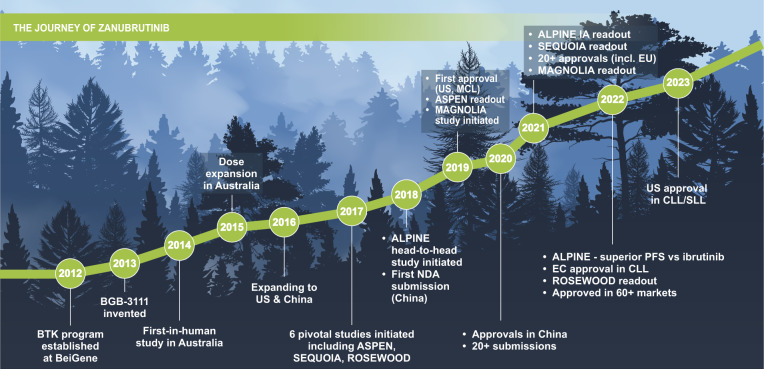
Key milestones in the development of zanubrutinib. BTK Bruton tyrosine kinase, CLL chronic lymphocytic leukemia, EC European Commission, EU European Union, IA interim analysis, MCL mantle cell lymphoma, NDA new drug application, PFS progression-free survival, SLL small lymphocytic lymphoma, US United States.

Since 2019, there has been a continuous flow of study readouts, publication of positive results, presentations in major congresses, and approvals relating to zanubrutinib. As of May 2023, zanubrutinib has been approved in multiple indications in more than 60 countries and regions. Zanubrutinib was initially approved in 2019 in the United States for patients with previously treated MCL, followed by approvals in China in 2020, and 21 additional approvals in 2021 (Fig. 6) [57].

### Mantle cell lymphoma

In November 2019, the FDA granted zanubrutinib accelerated approval for the treatment of patients with MCL who have received at least 1 prior therapy, based on the results from the BGB-3111-AU-003 (NCT02343120) and the BGB-3111-206 (NCT03206970) studies. The former was the first-in-human, dose-escalation study of zanubrutinib in various B-cell malignancies, including 37 patients with relapsed or refractory (R/R) MCL and 11 patients with treatment-naive (TN) MCL. Patients with R/R MCL had an overall response rate (ORR) of 87% with a complete response (CR) rate of 30% at a median follow-up of 19.4 months; the median PFS was 17.3 months. In this same study, patients with TN MCL had an ORR of 82%, with 27% of patients achieving CR [62, 63]. The latter study was conducted in China and evaluated zanubrutinib in 86 patients with R/R MCL, with a resultant ORR of 83.7% and 77.9% of patients achieving CR. Median PFS was 33 months, and median OS was not reached after 35.3 months of follow-up [64, 65]. A pooled safety analysis of both studies reported low rates of atrial fibrillation (1.8%; 0.9% grade ≥3) and a 12.5% discontinuation rate due to adverse events [66].

Acalabrutinib was approved in October 2017 for patients with R/R MCL [67] based on the results of 124 patients included in the single-arm ACE-LY-004 study (NCT02213926) [68]. The ORR and CR rate were 81% and 40%, respectively, after a median follow-up of 15.2 months, and the estimated 12-month PFS was 67%. The most frequent grade ≥3 adverse events were neutropenia, anemia, and pneumonia; no cases of atrial fibrillation were reported. Discontinuation rate due to adverse events was 7% [68].

### Waldenström macroglobulinemia

Ibrutinib has proven to be beneficial for patients with WM, but ibrutinib-related adverse events and reduced efficacy in patients with *CXCR4* mutations limit its use for that subset [69]. Preclinical and early-phase results of zanubrutinib gave researchers the confidence to run 2 head-to-head phase 3 studies against ibrutinib. One of them, the ASPEN study (NCT03053440) in patients with WM, formed the basis for the FDA approval on August 31, 2021, of zanubrutinib in this indication. Patients with the mutation of *MYD88* L265P were randomized to zanubrutinib (*n* = 101) or ibrutinib (*n* = 98) in cohort 1, and patients with *MYD88* wild-type WM (*N* = 28) received zanubrutinib in a nonrandomized arm (cohort 2). In cohort 1, after 44.4 months of median follow‑up, aggregated CR and VGPR rates were 36.3% versus 25.3% for zanubrutinib and ibrutinib, respectively; although not statistically significantly different, hazard ratio estimates favored zanubrutinib in cohort 1 (PFS: HR 0.63, 95% CI 0.36–1.12) [70]. These results should be analyzed in the context of the stratification methodology used for *CXCR4* mutations, which underreported the number of patients with *CXCR4* WHIM mutations. When using a more sensitive next-generation sequencing assay, an imbalance favoring ibrutinib (with 22% of patients with *CXCR4* WHIM mutations vs 33% in the zanubrutinib group) was observed. This impacted the comparison of responses between the 2 groups because *CXCR4* WHIM mutations are associated with lower VGPR rates. Median PFS and OS have not been reached. PFS rates at 42 months were 78.3% for zanubrutinib and 69.7% for ibrutinib. In cohort 2, patients with *MYD88* wild-type WM had a 65% response rate, including 1 CR [70].

ASPEN was the first head-to-head comparison of 2 BTK inhibitors to be reported and gave a unique opportunity to examine the different toxicities of first- and second- generation BTK inhibitors. In this comparison, zanubrutinib was associated with fewer adverse events leading to dose reductions, treatment discontinuations, and deaths, compared to ibrutinib. In addition, atrial fibrillation and bleeding rates were lower in the zanubrutinib arm at all time intervals compared to that of ibrutinib, and hypertension rates trended lower over time (*P* = 0.16). Even though neutropenia was more frequent in the zanubrutinib group, the rate of infections was similar (any grade) or higher (grade ≥3) in the ibrutinib group [70]. Another earlier study of patients with WM was BGB-3111-AU-003, reporting ORR and CR + VGPR rates of 93.9% and 51%, respectively, a 24-month PFS rate of 76.2%, and a 24-month OS of 91.5% in 49 patients with R/R WM. Among the 24 patients with TN WM, all had a response and 33.3% achieved CR + VGPR, and the 24-month PFS and OS rates were 91.5% and 100%, respectively [71]. Finally, the phase 2 trial BGB-3111-210 (NCT03332173) included 44 high-risk patients with R/R WM treated with zanubrutinib. The study reported a 33% CR + VGPR rate and a 24-month PFS of 61%, with most grade ≥3 adverse events being hematologic with no reports of atrial fibrillation or flutter. These results were consistent across patient subgroups including patients with *MYD88* L265P and/or mutation of *CXCR4* WHIM [72].

### Marginal zone lymphoma

Zanubrutinib received accelerated approval from the FDA on September 14, 2021, for patients with R/R MZL who have received at least 1 anti-CD20–based regimen. The MAGNOLIA study (NCT03846427), a single-arm phase 2 study, showed an ORR of 74% with a 24% CR rate at a median 10.7 months of follow-up. The 2-year survival rate was 86% in patients with MZL, and responses were observed in all MZL subtypes and in difficult-to-treat disease subgroups. One of 68 patients had grade ≥3 atrial flutter, and 2 patients discontinued zanubrutinib due to adverse events [73].

### Chronic lymphocytic leukemia and small lymphocytic lymphoma

On January 19, 2023, zanubrutinib received FDA approval in CLL/SLL based on the second head-to-head study versus ibrutinib (ALPINE) and the SEQUOIA study. The ALPINE study (NCT03734016), which included patients with R/R CLL/SLL who were randomized to zanubrutinib (*n* = 327) or ibrutinib (*n* = 325), demonstrated superiority of zanubrutinib over ibrutinib in ORR and PFS. The ORR (CR, nodular partial response, or partial response) was significantly (*P* = 0.0133) higher with zanubrutinib (80.4%) versus ibrutinib (72.9%), and the PFS was significantly (*P* = 0.002) longer with zanubrutinib versus ibrutinib, with a hazard ratio of 0.65 (95% CI 0.49–0.86). This difference was consistent across patient subgroups, including patients with deletion of the 11q22.3 chromosomal region, or 17p deletion/mutation of tumor-protein p53 [74]. In the high-risk population with del(17p13.1)/*TP53* mutation, the superior PFS benefit with zanubrutinib remained, with a hazard ratio of disease progression or death of 0.53 (95% CI 0.31‒0.88) by investigator assessment [74]. Zanubrutinib safety/tolerability profile was also improved over ibrutinib with fewer adverse events leading to treatment discontinuation and fewer cardiac events, including fewer cardiac events leading to discontinuation or death.

Acalabrutinib is the only other second-generation BTK inhibitor to be compared directly with ibrutinib in a clinical study. The ELEVATE-RR study (NCT02477696) was a noninferiority study of acalabrutinib versus ibrutinib in patients with previously treated CLL who had del(17p13.1) and/or del(11q22.3). In this study, acalabrutinib met its primary endpoint of noninferiority with a median PFS of 38.4 months in both arms (HR 1.0; 95% CI 0.79‒1.27) [75]. Although cross-trial comparison is difficult owing to various factors (e.g., different patient populations) and interpretation should be made with caution, it should be noted that unlike zanubrutinib, which was observed to have improved benefits over ibrutinib in the high-risk del(17p13.1)/*TP53* mutation subgroup, this was not seen with acalabrutinib.

In the SEQUOIA study (NCT03336333) of zanubrutinib versus BR in patients with TN CLL/SLL, patients without del(17p13.1) were randomly assigned to zanubrutinib (*n* = 241) or BR (*n* = 238); those with del(17p13.1) CLL/SLL were assigned to zanubrutinib in a different arm (*n* = 111). The ORR was 94.6% and 85.3% in the zanubrutinib and BR arms, respectively, including 7% and 15% of patients who achieved CR. Patients treated with zanubrutinib showed improved PFS versus those treated with BR (HR 0.42; 95% CI 0.28–0.63; *P* < 0.0001), and PFS was consistently longer with zanubrutinib in most subgroups such as older patients, patients with high-risk disease, patients with Binet stage C disease, bulky disease, and presence of unmutated *IGHV*, or del(11q22.3). Among patients with del(17p13.1) CLL/SLL, 24-month PFS and 24-month OS rates were 89% and 93.6%, respectively. Treatment discontinuations, dose reductions, and adverse events leading to treatment discontinuation were less frequent in the zanubrutinib arm [76]. With the longer follow-up in SEQUOIA, the estimated 42-month PFS rates were 82% for the zanubrutinib arm and 50% for the BR arm, and the 42-month OS rates were 89% and 88%, respectively. The tolerability profile of zanubrutinib remained acceptable, including low rates of atrial fibrillation [77].

In the ELEVATE-TN study (NCT02475681), the clinical effects of acalabrutinib, with or without obinutuzumab, were compared against chlorambucil with obinutuzumab alone in patients with TN CLL [78]. Acalabrutinib, as a single agent or in combination with obinutuzumab, showed improved PFS over obinutuzumab-chlorambucil chemoimmunotherapy. The side-effect profile was acceptable and consistent with those of earlier results and other second-generation BTK inhibitors.

## The future: ongoing research with zanubrutinib

As of May 2023, zanubrutinib has been studied in a broad global clinical development program in more than 3900 patients in 35 clinical studies across 28 countries, and these numbers keep growing (Table 2).

**Table 2:** Zanubrutinib studies.

Indication	Study and National Clinical Trial No.	Line	Phase	Interventions (N/n)	mFU, mo	Key efficacy endpoints	Discontinuations due to AEs	Status
B-cell malignancies	BGB-3111-1002 [60] *NCT03189524*	R/R	1	Zanu (44)	31.5	ORR: 52.3%; CR: 18.2%	4.50%	Completed
	BGB-3111-111 *NCT04172246*	R/R	1/2	Zanu (53)	NR	NR	NR	Active, not recruiting
	BGB-3111-LTE1 *NCT04170283*	TN and R/R	3	Zanu vs Zanu + T (500)	NR	NR	NR	Enrolling by invitation
B-cell lymphoma^a^	BGB-3111-215 *NCT04116437*	R/R	2	Zanu (90)	NR	NR	NR	Recruiting
CLL/SLL	BGB-3111-AU-003 [53] *NCT02343120*	TN and R/R	1/2	Zanu (94)	13.7	ORR: 96.2%; CR: 2.6%	2.10%	Completed
	BGB-3111-GA101 [98] *NCT02569476*	R/R	1/2	Zanu (25)	29	ORR: 92%; CR: 28%	9%	Completed
	BGB-3111-GA101 [98] *NCT02569476*	TN	1/2	Zanu (20)	29	ORR: 100%; CR: 30%		Completed
	BGB-3111-205 [99] *NCT03206918*	R/R	2	Zanu (91)	15.1	ORR: 84.6%; CR: 3.3% 12-mo PFS: 87.2%	9%	Completed
	ALPINE (BGB-3111-305) [56] *NCT03734016*	R/R	3	Zanu (327) vs Ibr (325)	29.6	ORR: Zanu: 80.4% vs Ibr: 72.9% CR: Zanu: 4% vs Ibr: 2.5% 24-mo PFS: Zanu: 78.4% vs Ibr: 65.9%	Zanu: 15.4% Ibr: 22.2%	Active, not recruiting
	SEQUOIA (BGB-3111-304) [76] *NCT03336333*	TN	3	Zanu (241) vs BR (238)	26.2	ORR: Zanu: 94.6% vs BR: 85.3% CR: Zanu: 7% vs BR: 15% 24-mo PFS: Zanu: 85.5% vs Ibr: 69.5%	Zanu: 8% Ibr: 14%	Active, not recruiting
DLBCL	BGB-3111-207 [100] *NCT03145064*	R/R	2	Zanu (41)	6.8	ORR: 29.3%; CR: 17.1% mPFS 2.8 mo	9.80%	Completed
	BGB-3111-213 [100] *NCT03520920*	R/R	2	Zanu + R (20)	10.3	ORR: 35%; CR: 5%	0%	Completed
	BGB-3111-A317 [100] *NCT02795182*	R/R	1/2	Zanu + T (27)	4.1	ORR: 37%; CR: 15%	13%	Completed
FL	BGB-3111-213 [100] *NCT03520920*	R/R	2	Zanu + R (21)	NR	NR	NR	Completed
	BGB-3111-GA101 [98] *NCT02569476*	R/R	1/2	Zanu (36)	20	ORR: 72%; CR: 39%	3%	Completed
	ROSEWOOD (BGB-3111-212) *NCT03332017*	R/R	2	Zanu + Obi vs Obi	NR	NR	NR	Active, not recruiting
	MAHOGANY (BGB-3111-308) *NCT05100862*	R/R	3	Zanu + Anti-CD20 vs Len + R	NR	NR	NR	Recruiting
MCL	BGB-3111-AU-003 [53] *NCT02343120*	R/R	1/2	Zanu (37)	19.4	ORR: 87%; CR: 30% PFS: 17.3 mo	25%	Completed
	BGB-3111-AU-003 [53] *NCT02343120*	TN	1/2	Zanu (11)	8.3	ORR: 82%; CR: 27% PFS: NR	21%	Completed
	BGB-3111-206 [65] *NCT03206970*	R/R	2	Zanu (86)	35.3	ORR: 84%; CR: 78% PFS: 33 mo	9%	Completed
	BGB-3111-306 *NCT04002297*	TN	3	Zanu + R vs BR	NR	NR	NR	Recruiting
	CHESS *NCT04624958*	TN	2	Zanu + R followed by R-DHAOx	NR	NR	NR	Recruiting
MZL	MAGNOLIA (BGB-3111-214) [101] *NCT03846427*	R/R	2	Zanu (68)	28	ORR: 68%; CR: 26% 24-mo PFS: 71%	7%	Completed
	BGB-3111-213 [100] *NCT03520920*	R/R	2	Zanu (5)	10.3	ORR: 60%; CR: 20% 12-mo PFS: 75%	0%	Completed
	MAHOGANY (BGB-3111-308) *NCT05100862*	R/R	3	Zanu + Anti-CD20 vs Len + R	NR	NR	NR	Recruiting
WM	BGB-3111-AU-003 [53] *NCT02343120*	R/R	1/2	Zanu (49)	35.8	CR+VGPR: 51% 24-mo PFS: 76.2%	14%	Completed
	BGB-3111-AU-003 [53] *NCT02343120*	TN	1/2	Zanu (24)	23.5	CR+VGPR: 33.3% 24-mo PFS: 91.5%	13%	Completed
	BGB-3111-210 [100] *NCT03332173*	R/R	2	Zanu (44)	33	CR+VGPR: 33% 24-mo PFS: 61%	14%	Completed
	ASPEN (BGB-3111-302) [70] *NCT03053440*	TN and R/R	3	Zanu (101) vs Ibr (98)	44.1	CR+VGPR: Zanu: 36.3% vs Ibr: 25.3% 42-mo PFS: Zanu: 78.3% vs Ibr: 69.7%	Zanu: 8.9% Ibr: 20.4%	Completed

*AE* adverse event, *B* bendamustine, *CLL* chronic lymphocytic leukemia, *CR* complete response, *DLBCL* diffuse large B-cell lymphoma, *FL* follicular lymphoma, *Ibr* ibrutinib, *Len* lenalidomide, *MCL* mantle cell lymphoma, *mFU* median follow-up, *mo* month, *mPFS* median progression-free survival, *MZL* marginal zone lymphoma, *NR* not reached, *Obi* obinutuzumab, *ORR* overall response rate, *PFS* progression-free survival, *R* rituximab, *R-DHAOx* rituximab, dexamethasone, cytarabine, and oxaliplatin, *R/R* relapsed/refractory, *SLL* small lymphocytic lymphoma, *T* tislelizumab, *TN* treatment naive, *VGPR* very good partial response, *vs* versus, *WM* Waldenström macroglobulinemia, *Zanu* zanubrutinib.

^a^Intolerant to ibrutinib or acalabrutinib.

Because of its lower toxicity profile, zanubrutinib is also being studied in an exploratory phase 2 study (NCT04116437) in patients with B-cell malignancies who have been treated and are intolerant to ibrutinib or acalabrutinib. This study included 67 patients with B-cell malignancies who became intolerant to ibrutinib, acalabrutinib, or both. Most ibrutinib- or acalabrutinib-related toxicities did not recur or recurred at a lower severity with zanubrutinib. In addition, disease control was maintained by 94% of patients. The results of this study highlight the safety and efficacy of zanubrutinib in this group of patients with otherwise limited treatment options and potentially extend the opportunity for clinical benefit within the drug class of covalent BTK inhibitors [49].

To further evaluate therapy outcomes and benefit a greater number of patients, additional studies were designed. Ongoing studies include a phase 3 study (NCT04002297) of newly diagnosed patients with MCL (zanubrutinib + rituximab vs BR), the phase 2 study CHESS (NCT04624958) in patients with previously untreated MCL (zanubrutinib + rituximab vs rituximab, dexamethasone, cytarabine and oxaliplatin), and the phase 3 study MAHOGANY (NCT05100862) in patients with R/R MZL (zanubrutinib + rituximab vs lenalidomide + rituximab). The MAHOGANY study also includes patients with follicular lymphoma and will be the phase 3 confirmatory study for this indication. The phase 2 study ROSEWOOD (NCT03332017) tested the zanubrutinib + obinutuzumab combination versus obinutuzumab monotherapy in patients with R/R follicular lymphoma. Results of the ROSEWOOD study with a median follow-up of 20.2 months showed that median PFS was 28 months for the combination and 10.4 months for obinutuzumab monotherapy, with an HR of 0.50 (95% CI 0.33‒0.75); *P* = 0.0007 [79].

Accumulation of data from patients treated with zanubrutinib has provided robust insights on its overall safety and tolerability profile. Zanubrutinib typically is well tolerated, with generally mild-to-moderate adverse events that are usually manageable and not associated with frequent treatment discontinuations. Pooled data from 10 clinical trials in B-cell malignancies, including 1550 patients treated with zanubrutinib, showed low treatment discontinuation rates due to adverse events [80, 81]. The prevalence of adverse events of special interest such as infections, hemorrhage, neutropenia, thrombocytopenia, hypertension, anemia, secondary malignancies, and atrial fibrillation/flutter tend to remain constant or decrease over time [81]. In addition, zanubrutinib appears to be generally associated with fewer cardiovascular adverse events compared with ibrutinib. Based on pooled data from the ASPEN and ALPINE studies, the exposure-adjusted incidence rate of cardiovascular adverse events was significantly lower for zanubrutinib compared to ibrutinib, including atrial fibrillation (*P* < 0.0001), and symptomatic ventricular arrhythmias (*P* = 0.0028) [82].

The promising safety profile of zanubrutinib allows for the exploration of new combinations with agents that may provide synergistic effects. New studies are ongoing of zanubrutinib in combination with other targeted therapies, including BCL-2 inhibitors, PI3K inhibitors, chimeric antigen receptor (CAR) T-cell therapy, and checkpoint inhibitors. The phase 2 ZANU-VEN study (NCT05168930) is assessing the zanubrutinib + venetoclax combination in CLL; the zanubrutinib + BGB-10188 combination is being tested in B-cell malignancies in a phase 1/2 study (NCT04282018); and the triplet combination of zanubrutinib + venetoclax + obinutuzumab is being studied in patients with CLL in a phase 2 study (BoVEN: NCT03824483). This study reported deep molecular responses with a median follow-up of 40 months, with 96% and 92% of patients achieving negative minimal residual disease in peripheral blood and bone marrow, respectively, and good tolerability. Patients with negative minimal residual disease by flow cytometry (MRD-FC) had a MRD-FC free survival of 29.8 months [83].

Some evidence suggests that the combination of BTK inhibitors with CAR T-cell therapies may increase CAR T-cell expansion, viability, and engraftment during the manufacturing process, and enhance CAR T-cell activation and effector function [84–87]. An ongoing phase 3 clinical trial (NCT05020392) in China is assessing the efficacy and safety of anti-CD19 CAR T-cell therapy with concurrent BTK inhibitor (ibrutinib, zanubrutinib, or orelabrutinib) in patients with R/R B-cell malignancies, with expected results by the end of 2023. Results published highlight the clinically significant relevance of zanubrutinib in the treatment armamentarium of B-cell malignancies. Confidence in the benefits of zanubrutinib is exemplified by its inclusion in international treatment guidelines for CLL and non-Hodgkin lymphoma [88, 89].

Despite the benefits of BTK inhibitors in the treatment of B-cell malignancies, some unmet needs require further research. Continuous use of BTK inhibitors may lead to the acquisition of mutations in the BTK binding site (cysteine 481) or in other components in the signaling pathway (such as PLCG2). New noncovalent BTK inhibitors that do not depend on cysteine 481 (e.g., pirtobrutinib) are under development, with the hope of overcoming resistance mechanisms [90]. Enrichment in mutations that may confer resistance have been reported after treatment with specific BTK inhibitors. For example, the mutation leucine 528 substitution to tryptophan has been detected mainly in patients treated with zanubrutinib but not ibrutinib. Moreover, this mutation has shown cross resistance with pirtobrutinib [91]. These results highlight the importance to further investigate resistance mechanisms and the impact on different treatment choices. Other strategies targeting BTK include specific protein degraders, including NX-2127 which has been shown to degrade BTK independently of *C481* mutations [92, 93]. These new strategies may help reduce resistance mutations and provide therapeutic alternatives upon disease progression in patients treated with covalent BTK inhibitors in earlier lines of treatment.

Moreover, AbbVie recently announced the intention to withdraw ibrutinib from the United States market for R/R MCL and R/R MZL based on results of phase 3 confirmatory studies, necessitating alternative therapies for these patients [94].

In conclusion, this review of the history of zanubrutinib highlights the importance of multidisciplinary collaborative research, from early chemical research to clinical studies, and provides an example of how progress is incremental. Despite remarkable efficacy demonstrated with first-generation compounds, there is always room for improvement in molecular design and resultant patient care.

## Data Availability

This article file has no independent data.
