# Correction: Zanubrutinib: past, present, and future

**DOI:** 10.1038/s41408-023-00926-3

**Published:** 2023-10-02

**Authors:** Constantine S. Tam, Javier L. Muñoz, John F. Seymour, Stephen Opat

**Affiliations:** 1 Alfred Hospital and Monash University, Melbourne, VIC Australia; 2 Mayo Clinic, Phoenix, AZ USA; 3 Peter MacCallum Cancer Centre, Royal Melbourne Hospital & University of Melbourne, Melbourne, VIC Australia; 4 Monash Health and Monash University, Clayton, VIC Australia

**Keywords:** Drug development, Drug development

Correction to: *Blood Cancer Journal* 10.1038/s41408-023-00902-x, published online 11 September 2023

In the sentence beginning ‘Zanubrutinib achieved 100% peripheral blood...’ in this article, the text ‘160 mg twice daily (BID) or 320 mg once daily (QD), respectively’ should have read ‘320 mg once daily (QD) or 160 mg twice daily (BID), respectively.’.

The original article has been corrected.

